# Cellulose-Based Sorbents: A Comprehensive Review of Current Advances in Water Remediation and Future Prospects

**DOI:** 10.3390/molecules29245969

**Published:** 2024-12-18

**Authors:** Akmaral Darmenbayeva, Reshmy Rajasekharan, Bakytgul Massalimova, Nessipkhan Bektenov, Raushan Taubayeva, Karlygash Bazarbaeva, Musrepbek Kurmanaliev, Zhazira Mukazhanova, Aisha Nurlybayeva, Kamila Bulekbayeva, Aisulu Kabylbekova, Aisulu Ungarbayeva

**Affiliations:** 1 Department of Chemistry and Chemical Technology, M.Kh. Dulaty Taraz University, Taraz 080000, Kazakhstan; raushan.taubaeva@mail.ru (R.T.); rustem_ergali@mail.ru (A.N.); nurhat2000@mail.ru (K.B.); 2 Department of Science and Humanities, Providence College of Engineering, Kerala 689122, India; 3 Department of Chemistry and Chemical Engineering, M. Kozybayev North Kazakhstan University, Petropavlovsk 150000, Kazakhstan; bkmasalimova@ku.edu.kz; 4 Department of Chemistry, Abay Kazakh National Pedagogical University, Almaty 050010, Kazakhstan; bektenbna@gmail.com; 5 Department of Biotechnology and Microbiology, Eurasian National University, Astana 140002, Kazakhstan; karlygash.ba@mail.ru; 6 Department of Chemistry and Chemical Technology, Almaty Technological University, Almaty 050002, Kazakhstan; mkk@mail.ru; 7 Higher School of IT and Natural Sciences, S. Amanzholov East Kazakhstan University, Ust-Kamenogorsk 070010, Kazakhstan; mukazhanovazhb@mail.ru; 8 Department of Chemistry, Biology and Physical Education, Miras University, Shymkent 160012, Kazakhstan; aika_kabil@mail.ru; 9 Department of Chemistry, Biology and Ecology, Central Asian Innovation University, Shymkent 160000, Kazakhstan; ungarbaeva.aysulu@mail.ru

**Keywords:** cellulose-based sorbents, methods of obtaining sorbents, sorption properties, cellulose modification, water purification, ecological sorbents, ion exchange, adsorption, green technologies, complexation

## Abstract

Cellulose-based sorbents are promising materials for wastewater treatment due to their environmental friendliness, biodegradability, and high sorption capacity. This paper presents an overview of cellulose modification methods, including carboxylation, amination, oxidation, graphene, and plasma treatments, as well as combined approaches. Their effect on key physicochemical properties, such as porosity, morphology, and chemical stability, is considered. Examples from the literature confirm the effectiveness of modified cellulose sorbents in removing heavy metal ions and organic pollutants from wastewater. The analysis shows that combined methods allow for creating materials with improved characteristics that are resistant to extreme operating conditions. The main advantages and disadvantages of cellulose sorbents, as well as challenges associated with their scalability and cost-effectiveness, are discussed. The paper emphasizes the importance of further research to advance these materials as a key element of sustainable water treatment technologies.

## 1. Introduction

Water purification from pollutants is one of the priority tasks of modern society, especially in the context of industrial growth and urbanization, accompanied by an increase in wastewater volumes. Traditional purification methods, including coagulation, flotation, filtration and the use of activated carbon, have proven their effectiveness at a basic level. However, their use is associated with high energy intensity, significant operating costs and limited ability to remove specific pollutants, such as heavy metals and complex organic substances. These limitations have stimulated the development of more sustainable and efficient water purification technologies.

Modern approaches include membrane processes, synthetic polymer sorbents, and nanomaterials, which demonstrate high efficiency, but are often associated with problems, such as biodegradability and cost. In this regard, there is a growing interest in the use of natural materials, such as cellulose, as a basis for the creation of environmentally friendly sorbents. Cellulose, due to its availability, low cost, and the possibility of modification, is a unique material for the development of efficient and sustainable water purification technologies. The functionalization of cellulose allows for a significant increase in its adsorption properties, providing selective removal of pollutants, including heavy metals, dyes, and organic compounds. In addition, the integration of cellulose with nanotechnology and the development of multifunctional “smart” materials open up prospects for the creation of a new generation of sorbents capable of adapting to various operating conditions and simultaneously extracting valuable substances from wastewater.

It is known that cellulose-based sorbents have been the subject of many studies. However, unlike most studies that focus only on individual aspects, such as chemical modification methods or practical use of cellulose for water purification, this paper attempts to combine all stages: from preparation of the starting material to assessment of its durability and efficiency under real operating conditions.

A unique feature of this review is a deep comparative analysis of cellulose functionalization methods, including chemical, physical, and biological approaches, with a detailed description of their effect on the adsorption properties of the material. In addition, the review focuses on a wide range of applications of cellulose sorbents: from water purification and medicine to agrochemistry and energy, which allows for a demonstration of their versatility. Thus, this paper not only systematizes the current knowledge but identifies gaps, highlighting the importance of further research in this area.

## 2. Cellulose as a Basis for Creating Sorbents

Cellulose is a linear polysaccharide composed of repeating β-d-glucose units linked by β-(1→4)-glycosidic bonds. It has a complex network of hydrogen bonds, which determines its high mechanical strength and resistance to aggressive chemicals [1,2,3]. The presence of crystalline and amorphous regions is a key factor that affects the physicochemical properties of cellulose, such as sorption capacity and reactivity. Crystalline regions are less reactive due to the high density of bonds, while amorphous zones of cellulose, due to the greater mobility of the polymer chains, promote better interaction with water molecules and dissolved substances [4].

Cellulose also has a high degree of polydispersity and can be isolated from various plant sources, including wood, cotton, and algae. Its unique structure allows for the creation of a variety of forms, such as cellulose fibers, microfibrils, and nanocrystals, which exhibit different physical and chemical properties [5].

The main sorption capacity of cellulose is due to the presence of multiple hydroxyl groups (-OH), which can interact with various ions and molecules. These groups can be chemically modified to improve sorption properties. For example, cellulose can be carboxylated or sulfonated, which increases the number of active sites for binding heavy metal ions and organic pollutants [6,7,8]. Modified cellulose shows improved sorption properties due to the expansion of the range of reactive functional groups. For example, carboxylated cellulose effectively adsorbs lead (Pb^2+^), cadmium (Cd^2+^), and copper (Cu^2+^) ions, as shown by of O’Connell et al. [9], where modification of the cellulose surface with carboxyl groups created additional binding sites for metal ions. Further improvement of chemical methods for modifying cellulose opens up prospects for its use as a sorbent in water purification and environmental protection systems.

Cellulose nanocrystals (CNC) and nanofibrillated cellulose (CNF) have attracted considerable attention due to their high specific surface area and excellent mechanical properties. Cellulose nanocrystals isolated from the crystalline regions of cellulose have a high degree of ordering, which makes them stable and effective for creating sorbents with high sorption capacity [10].

Nanocrystalline cellulose, compared with macroscopic cellulose, has better dispersion in solutions, which increases its active surface area and the ability to sorb pollutant molecules, such as organic compounds and metals. These nanomaterials can be effectively applied in wastewater treatment and toxic substances removal [11,12].

Thus, cellulose as a base for sorbents has a number of significant advantages. Firstly, it is a renewable and affordable resource, which makes it attractive for mass use in environmentally friendly technologies. Secondly, cellulose is a biodegradable material, which reduces environmental risks during its disposal and makes such sorbents ideal for use in wastewater and soil treatment [13].

Another important advantage of cellulose sorbents is their ability to regenerate. Cellulose sorbents can be reused many times, which significantly reduces their operating costs and makes them cost-effective for wide industrial use [14].

The key physical and chemical properties of cellulose, as well as their importance for sorption, are given in Table 1.

Thus, cellulose is a unique material for creating sorbents due to its availability, environmental friendliness, chemical modifiability, porosity, and mechanical strength. These properties allow it to effectively sorb a wide range of pollutants, which makes it promising for use in environmental technologies for cleaning water, air, and wastewater.

## 3. Methods of Pre-Treatment of Cellulose

Methods of pre-treatment of cellulose play a key role in improving its sorption properties and the possibility of application in various industries, such as ecology, medicine, and pharmaceuticals. These methods are aimed at destroying the crystalline structure of cellulose, increasing its porosity, availability of active groups, and improving interaction with pollutants.

One of the most effective methods is steam explosion [22,23], which involves treating cellulose with saturated steam under high pressure, and then rapidly reducing the pressure. This process destroys the cellular structure, increasing the availability of hydroxyl groups and improving the sorption characteristics of the material. It is especially suitable for processing lignocellulosic materials and is considered environmentally friendly, since it does not require the use of chemical reagents.

The mechanical activation method [24,25], in which cellulose is ground using mills or other mechanical devices, helps to increase the surface area and porosity of the material. This makes cellulose more accessible for the adsorption of pollutants. However, this method requires significant energy costs and is not always effective in breaking down the crystalline structure of cellulose.

Ultrasound treatment is a technology that uses ultrasound waves to break down cellulose fibers [26,27,28]. This method helps to increase the surface area and accessibility of reactive groups. However, its scope of application is limited, and fine-tuning of ultrasound parameters is necessary to achieve the best results.

Thermochemical treatment [29], which combines thermal action with chemical reagents, can significantly improve the properties of cellulose. This method is effective in removing lignin and hemicellulose, as well as increasing the hydrophilicity of the material, which expands its application areas.

Today, one of the most common methods of pre-treatment of cellulose is alkaline treatment [30,31,32,33]. This method has become widespread due to its high efficiency, low cost, and ability to be scaled up for industrial use. In addition, alkaline treatment has good scalability, which allows it to be used in large volumes, and is suitable for various types of cellulose, including wood, flax fiber, cotton, and other plant materials. This makes the method universal and in demand in various industries. Despite the use of chemical reagents, alkaline treatment is more environmentally friendly compared to acid and thermal methods, as it is accompanied by fewer by-products and is safer. However, to achieve cellulose with special properties, or in cases of complex applications, alkaline treatment is often combined with other methods, such as microwave [34] or ultrasound treatment [35]. This can significantly improve the sorption characteristics of cellulose, expanding its potential for various technological applications.

Thus, the choice of the cellulose pretreatment method depends on the specific requirements of the material and its end use. Each method has its own advantages and disadvantages, and in most cases a combination of several methods is required to achieve optimal results.

## 4. Methods for Obtaining Cellulose-Based Sorbents

The main methods for obtaining cellulose-based sorbents are related to improving its sorption properties through various chemical and physical modifications. These approaches provide adaptation of cellulose materials for various applications, including the removal of heavy metals, organic pollutants, and other toxic substances from water and air [36,37,38].

The number of functional groups (-OH groups) does not only change their chemical structure but changes when -OH groups are converted into sulfo, carboxyl, and other groups. For example, carboxymethyl cellulose (CMC) is widely used as a sorbent for the wastewater treatment of heavy metals due to its high affinity for metal cations [39]. The addition of amino groups also helps to improve the sorption properties, especially for metal ions and organic compounds [40].

Physical modification methods, such as thermal treatment, mechanical activation, or plasma modification, are aimed at improving the textural properties of cellulose, increasing porosity and surface area, which helps to increase the sorption capacity of the material. These methods are effective in improving the physical structure of cellulose without significant changes in its chemical composition. For example, thermal treatment can increase the porosity of cellulose, which allows it to be used to remove organic pollutants from water and air [41].

Combined approaches are also widely used in the development of cellulose sorbents. The use of both chemical and physical methods allows for the creation of multifunctional sorbents that have improved sorption capacity and resistance to aggressive operating conditions. These materials combine the advantages of various types of modifications, which allows for obtaining cellulose sorbents with high efficiency in removing a variety of pollutants [42].

Monitoring the properties of modified cellulose includes studying its mechanical, thermal, and sorption characteristics. This can be performed using methods such as scanning electron microscopy (SEM), atomic force microscopy (AFM), and dynamic mechanical spectroscopy (DMS) methods.

### 4.1. Chemical Modification of Cellulose

Chemical modification of cellulose involves the introduction of functional groups that increase its reactivity to pollutants, such as heavy metal ions, organic substances, and oils. These modifications are aimed at improving the sorption properties of cellulose, including its selectivity and stability.

#### 4.1.1. Carboxylation

The process of cellulose carboxylation includes several stages, each of which contributes to the transformation of cellulose hydroxyl groups into carboxyl groups (-COOH), which improves its sorption properties. In the first stage, cellulose is treated with an alkaline solution, usually sodium hydroxide (NaOH). The alkali interacts with the hydroxyl groups (-OH), which leads to their ionization and an increase in the reactivity of cellulose. This activation makes possible the further addition of chemical groups, in particular carboxyl ones. Activated cellulose becomes more accessible to reagents, which accelerates the process of chemical modification [43]. The carboxylation reaction then proceeds directly. One of the most common reagents for this step is monochloroacetic acid (MCA). The reaction of MCA with activated cellulose results in the replacement of hydroxyl groups with carboxyl groups. During this process, ether bonds are formed between the cellulose chain and the carboxyl group, resulting in the formation of carboxymethyl cellulose (CMC), the most well-known derivative of carboxylated cellulose [44]. After the chemical reaction of carboxylation is complete, the product must be purified from residual reagents and by-products. This usually involves washing the modified cellulose with water or aqueous solutions, which removes excess alkali and organic compounds. At this stage, additional material treatment may also be carried out to improve its physicochemical properties, such as increasing porosity or strength [45].

The degree of cellulose modification can be controlled by changing the reaction conditions. For example, increasing the concentration of monochloroacetic acid or increasing the reaction time leads to an increase in the number of carboxyl groups on the cellulose surface. This parameter is important, since the sorption capacity of the material depends on it: with a high degree of carboxylation, the sorbent becomes more effective in removing metal cations, but excessive modification can lead to deterioration of mechanical properties [46]. Carboxylation is one of the most studied methods of chemical modification of cellulose, since it significantly increases its efficiency in sorption processes, especially in the removal of heavy metal ions and organic pollutants. This process remains a relevant area of research, since carboxylated cellulose continues to find new areas of application in ecology and industry.

#### 4.1.2. Amination

Cellulose amination is a chemical modification process in which amino groups (-NH_2_) are introduced into the cellulose structure. This process significantly improves the properties of cellulosic materials, making them more effective sorbents for the removal of metal ions and organic pollutants from aqueous solutions. The amination procedure involves several key steps, each of which plays an important role in obtaining the modified product. The first step involves the preparation of cellulose, which can be obtained from various sources, such as wood, cotton, or other plant fibers. The starting material must be purified from any impurities, such as lignin and hemicellulose, which is achieved by alkali treatment (e.g., using sodium hydroxide). This treatment improves the accessibility of the hydroxyl groups of cellulose, making them more reactive for subsequent amination [47].

In the second step, cellulose is activated using reagents such as dimethyl sulfoxide (DMSO) or pyridine, which promotes the ionization of hydroxyl groups and improves the reactivity of cellulose. This process creates active sites ready to react with the amine [48]. The key step of amination is the introduction of amino groups into the cellulose structure. Aliphatic or aromatic amines, such as ethanolamine or phenylglycine, are often used for this purpose. The amination reaction usually takes place under reaction conditions using a solvent that promotes the dissolution of both cellulose and the amine. The process can be carried out at elevated temperatures (usually between 60 and 100 °C) for several hours. It is important to control the ratio of cellulose to amine to avoid the formation of by-products and to ensure the desired degree of modification [49].

After the amination reaction is complete, the resulting modified product must be purified from any residual reagents and by-products. This is usually achieved by washing with distilled water or acid solutions to neutralize residual reaction substances. The product can also be dried at low temperature to prevent the destruction of new functional groups [50]. The degree of amination can be controlled by changing reaction conditions, such as temperature, reaction time, and amine concentration. This allows for an optimal balance between the amount of the amino groups introduced and the properties of the final product. For example, a high degree of amination can improve the sorption properties of a material, but can also lead to deterioration of its mechanical properties [51].

#### 4.1.3. Esterification

Cellulose esterification is a modification process that introduces ether groups into the cellulose structure, significantly improving its physicochemical properties and expanding its applications, including as sorbents for removing pollutants from aqueous solutions. The key step involves introducing ether groups into the cellulose structure. The most common etherifying agents are carboxylic acid anhydrides (e.g., acetic anhydride) or carboxylic acid chlorides (e.g., acetyl chloride). The esterification process usually takes place in a reaction mixture using a solvent and catalysts, such as pyridine. The reaction can be carried out at elevated temperatures (typically 40 to 80 °C) for several hours. Controlling the ratio of cellulose to the etherifying agent is important to achieve the optimal degree of modification and prevent the formation of by-products [52]. The degree of esterification, i.e., the amount of ester groups introduced, can be controlled by changing the reaction conditions, such as temperature, reaction time, and concentration of the esterifying agent. This allows the properties of the final product to be optimized for various applications, such as sorption and use in the pharmaceutical and cosmetic industries.

#### 4.1.4. Graphene Modification

Graphene modification of cellulose is a process that involves the introduction of graphene or graphene-like materials into the structure of cellulose to improve its physical and chemical properties. The first step, as in other methods, is to prepare cellulose. The next step is to obtain graphene or graphene-like materials. The most common methods for obtaining graphene include mechanical exfoliation, chemical vapor deposition (CVD), and chemical reduction of graphene oxides. Chemical reduction of graphene oxides is one of the most effective methods that allows for obtaining high-quality graphene using graphene oxide as a starting material [53]. The key step in graphene modification is the combined use of graphene and cellulose. Graphene can be incorporated into the cellulose matrix using various methods, including mechanical mixing, ultrasonic treatment, or chemical modification. In mechanical mixing, graphene nanoparticles and cellulose are mixed in a solvent, which promotes uniform distribution of graphene in the cellulose matrix [54].

Using ultrasonic treatment, graphene nanoparticles are dispersed in a solvent with cellulose, which improves their interaction and promotes the formation of a homogeneous mixture. Chemical modification can involve the reaction of graphene with functional groups of cellulose, which allows for the creation of a composite material with improved properties [55,56]. After completion of the modification reaction, the resulting composite material must be purified from residual reagents and by-products.

### 4.2. Physical Treatment of Cellulose Materials

Physical modification involves methods of treating cellulose to increase its porosity, specific surface area, and improve its adsorption properties. These methods usually do not change the chemical structure of cellulose but improve its physical properties.

#### 4.2.1. Mechanical Activation

Mechanical activation is a process that significantly changes the physicochemical properties of materials, such as cellulose, by applying mechanical forces. Mechanical activation can be carried out using different types of equipment, such as planetary or pebble mills. Mechanical activation consists of intense grinding and mixing of the material, which leads to the destruction of its structure and an increase in the contact surface. During the activation process, cellulose is exposed to impact and shear stresses, which leads to the formation of microcracks and improved accessibility of the functional groups for further reactions [57]. The duration of mechanical activation can vary depending on the desired characteristics of the final product. Typically, the activation time ranges from 30 min to several hours, depending on the type of equipment and process conditions [58]. Increasing the activation time can lead to a greater degree of grinding and improved sorption properties, but can also cause cellulose degradation, so process conditions must be carefully controlled.

#### 4.2.2. Thermal Treatment

Thermal treatment is an important step in the modification of cellulose to create sorbents, as it improves the physicochemical properties and sorption characteristics of the final product. This process can include various methods, such as pyrolysis, carbonization, and thermolysis, which provide significant changes in the structure and properties of cellulose. The first step of thermal treatment is the preparation of the starting material. In the next step, cellulose undergoes pyrolysis, which is the process of decomposition of organic material at high temperatures in the absence of oxygen. Pyrolysis leads to the formation of carbon material with high porosity, which can be used as an effective sorbent. The pyrolysis process is usually carried out at temperatures from 300 to 600 °C, and the treatment time can vary from a few minutes to several hours, depending on the desired properties of the final product [59]. Carbonization is another important step, which involves further thermal treatment of cellulose after pyrolysis. This process increases the carbon content of the final product, which improves its sorption properties. Carbonization usually occurs at higher temperatures, reaching 800–1000 °C. This process produces carbonized materials with a high surface area and porosity, making them suitable for use in wastewater treatment and other fields [60]. Thermolysis is a thermal treatment process in the presence of small amounts of oxygen or other reagents, which allows for control over the chemical composition and structure of the resulting material. Thermolysis can be used to modify cellulose to improve its sorption properties and resistance to moisture. Thermolysis temperatures are typically between 200 and 400 °C, and the process can take from a few hours to several days depending on the conditions [61].

#### 4.2.3. Plasma Treatment

This method involves the use of plasma, an ionized gas that can be created using a variety of technologies. There are several methods for generating plasma, including radio frequency (RF) and microwave (MW) plasma. RF plasma is most often used in laboratory settings because it allows for stable and uniform plasma distribution across the surface being treated. Microwave plasma is also widely used because it can treat larger areas and can be used for the continuous processing of materials [62]. Plasma processing involves exposing cellulosic materials to plasma for a specific period of time, which can vary depending on the desired characteristics of the final product. The processing time is typically between a few seconds and a few minutes. During plasma processing, the plasma interacts with the cellulose, which leads to the activation of functional groups on the surface of the material and the formation of new chemical bonds [63]. Plasma treatment results in modification of the cellulose surface, which may include the formation of oxygen-containing functional groups such as carboxyl and hydroxyl groups. These changes significantly increase the hydrophilicity of the material and its sorption characteristics, making it more effective as a sorbent for removing various pollutants from aqueous solutions. It is important to note that the depth of modification may depend on the treatment time and the type of plasma used [64].

#### 4.2.4. Radiation-Induced Modification

Radiation-induced modification is a promising approach to changing the structure and properties of cellulose to create effective absorbents for water purification and other applications. This method is based on the effect of ionizing radiation (gamma rays, X-rays, electron beams) on cellulose materials, which leads to the formation of active radicals and chemical reactions that improve sorption properties [65,66]. Under the influence of radiation, free radicals are formed in cellulose, which initiate reactions of destruction, cross-linking, or functionalization of the molecule. This leads to a change in the structure and chemical properties of cellulose. Radiation treatment allows for introducing various functional groups (carboxyl, sulfonic, amino groups) into the cellulose structure, increasing the sorption capacity and selectivity to heavy metal ions, dyes, and organic substances. Radiation can change the porous structure of cellulose, increasing its specific surface area and pore volume, which helps improve the access of pollutants to the active centers of the sorbent.

Techniques such as radiation-initiated graft polymerization (RIGP) are employed to alter the crystallinity of cellulose, enhancing its surface modification capabilities. The study successfully utilized radiation-initiated graft polymerization (RIGP) to modify cellulose surfaces, enhancing their capability for uranium adsorption. This method effectively reduced the crystallinity of cellulose, which is crucial for improving its application performance in adsorption. Amidoxime-modified cellulose/graphite oxide (Cel-AO/GO) composites were created, where graphite oxide served as a scaffold to compensate for the structural weakening of cellulose due to reduced crystallinity [67].

Thus, radiation-modified cellulose sorbents have emerged as effective materials for the adsorption of various heavy metals and radionuclides. The modification process enhances the sorbent’s surface properties, leading to improved adsorption capacities and kinetics. This overview will discuss the methods of modification, the performance of these sorbents, and their applications in environmental remediation.

### 4.3. Advantages and Disadvantages of the Methods of Cellulose Modification

The conclusions from an analysis of the literature emphasize the importance of using various cellulose modification methods to create effective sorbents designed to purify wastewater from heavy metal ions [68,69,70,71]. Table 2 describes the advantages and disadvantages of the main methods of cellulose modification.

Modifications such as carboxylation, amination, or graphene modification significantly improve the sorption properties of cellulose materials, increasing their ability to adsorb toxic ions such as Pb^2+^, Cd^2+^, Cr^3+^, and other pollutants. One of the key advantages of modifying cellulose sorbents is increasing their chemical stability and resistance to various external influences. As a result of such changes, cellulose becomes stronger and more durable, which is important to ensure long-term use of the sorbent in the wastewater treatment process. However, each individual method, such as carboxylation, amination, or plasma treatment, has its limitations, including a narrow focus on improving certain properties of the material or insufficient stability of sorbents under extreme operating conditions. To overcome these limitations and obtain materials with optimal characteristics, combined modification methods are increasingly used.

Combined methods allow for changing both the chemical composition of cellulose and its structure, providing a synergistic effect that significantly improves its functional properties. Combined methods often begin with the chemical modification of cellulose, which can include carboxylation, amination, or esterification reactions. After chemical modification, cellulose can be subjected to plasma treatment. This process creates active sites on the cellulose surface that can enhance its interaction with pollutants. Plasma modification helps to increase the porosity of the material and creates more active surfaces for the adsorption of molecules [73]. Thermal treatment, in turn, is used to stabilize the structure and create additional pores, which improves the mechanical properties of the material and makes it resistant to destruction under extreme operating conditions [74].

The combination of chemical modification with plasma or thermal treatment leads to the creation of sorbents that have high sorption activity in relation to various types of pollutants. For example, studies have shown that modified cellulose treated with plasma sorbs heavy metals significantly better compared to materials that have undergone only chemical modification [75]. This is due to the creation of additional microporosity and an increase in the number of active centers on the surface of cellulose.

## 5. Physicochemical Properties of Cellulose-Based Sorbents

**Figure 1:**
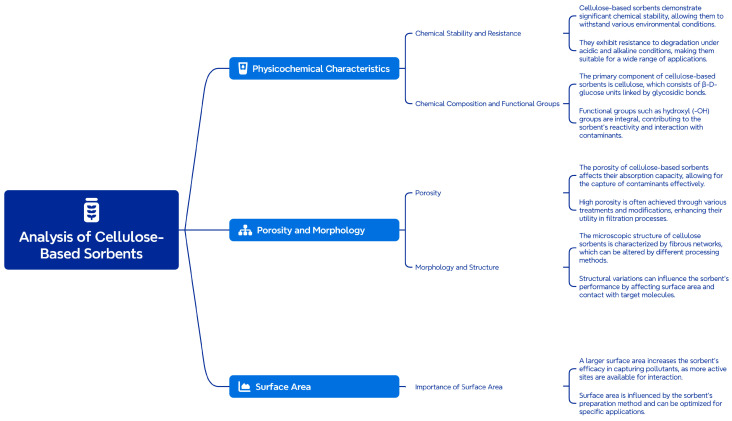
The main physicochemical characteristics of cellulose-based sorbents.

The physicochemical properties of cellulose-based sorbents play a key role in their effectiveness in purifying water, air, and other environments from pollutants. These properties depend on both the original cellulose structure and the modification methods used to improve the sorption characteristics. The most important physicochemical characteristics are shown in Figure 1.

### 5.1. Morphology and Structure

Cellulose consists of alternating crystalline and amorphous regions, and their ratio directly affects the sorption capacity of the material. The crystalline regions of cellulose are more ordered, which makes them less accessible for interaction with pollutants, while amorphous regions are more active in sorption processes. One paper [76] showed that an increase in the proportion of amorphous regions of cellulose due to mechanical activation helps to increase the material’s ability to adsorb water and organic pollutants.

Another example is presented in [77], where the modification of cellulose by oxidation led to the destruction of the crystalline structure, which increased the number of active sorption centers on the surface of the cellulose material. This contributed to the improvement of the adsorption of heavy metal ions, such as Cu (II) and Pb (II).

The morphology of cellulose sorbent particles can vary depending on the method of their preparation. For example, [78] described cellulose sorbents in the form of nanofibers, which demonstrated high adsorption properties due to the increased specific surface area and the presence of micropores. Fibers with a diameter of 50–200 nm showed high efficiency in removing heavy metal ions from wastewater. In addition, [79] considered an electrospinning method for obtaining cellulose sorbents in the form of nanofibers. Cellulose nanofibers with a diameter of about 100–300 nm demonstrated high adsorption properties for heavy metals, such as Pb (II) and Cd (II), due to their high surface area and multiple active functional groups.

### 5.2. Surface and Porous Characteristics

The surface area of the sorbent and the pore volume play an important role in the adsorption of both organic and inorganic substances. Studies have shown that increasing the specific surface area and creating mesopores and micropores improve the sorption properties of cellulose sorbents. In [80], it was shown that the modification of cellulose by thermal activation leads to an increase in the specific surface area of 124 m^2^/g, which significantly increases the sorption capacity for organic pollutants such as methylene blue and rhodamine.

When the average specific surface area of sorbents obtained by thermal activation reaches 50 m^2^/g [81], the authors found that such sorbents have a highly developed mesoporous structure that effectively adsorbs large organic molecules. Similar results were obtained in the work of Ibrahim and Eid [82], where plasma modification of cellulose fibers was demonstrated. Plasma treatment made it possible to create additional micropores on the surface of the fibers, which increased the total surface area to 80 m^2^/g and led to improved sorption characteristics for various organic dyes and heavy metals.

Cellulose sorbents can contain micropores (up to 2 nm), mesopores (2–50 nm) and macropores (more than 50 nm). Studies have shown that a combination of these pore types allows for efficient adsorption of both small and large molecules. For example, in [83], cellulose sorbents modified by the introduction of mesoporous structures were studied, which increased their ability to adsorb heavy metals such as Pb (II) and Cr (VI). The resulting materials had a specific surface area of 200 m^2^/g and a porosity of more than 80%, which improved the adsorption of both small metal ions and large organic molecules.

In [84], it was shown that cellulose sorbents modified using aerogels have a macroporous structure with a pore diameter of more than 100 μm, which were effective in the adsorption of large molecules of organic pollutants, such as oils and organic solvents.

Other studies have shown the effectiveness of creating cellulose aerogels with high porosity. The publication [85] described that cellulose aerogels with a specific surface area of about 250 m^2^/g demonstrated high sorption capacity for the removal of heavy metals from water. The pore volume of such materials was up to 90%, which ensured the rapid access of pollutants to the sorption centers.

In addition to porosity, an important factor is the modification of the sorbent surface. Examples of such modifications include carboxylation, amination, and the deposition of nanoparticles, which leads to the creation of additional sorption sites. One study [86] showed that the carboxylation of cellulose fibers increased the number of active sites on the surface, which contributed to the improvement of the sorption of metal ions (e.g., Cu (II) and Pb (II)). Surface modification improved both the specific surface area to 135 m^2^/g and the ion exchange efficiency.

Another example is presented in [87], where cellulose sorbents were modified with graphene nanoparticles to improve the sorption characteristics. The addition of graphene nanoparticles increased the surface area to 150 m^2^/g and simultaneously improved the conductivity of the material, which made it possible to remove both heavy metal ions and organic compounds.

Thus, the surface and porous structure of cellulose sorbents have a significant impact on their sorption characteristics. Improving these parameters by modifying materials, creating pores of different sizes, and increasing the specific surface area leads to a significant increase in their efficiency. The ability to effectively retain target pollutants, including heavy metals, organic dyes, and oils, should be at a level exceeding 100–200 mg/g for heavy metals and 500–1000 mg/g for organic dyes. The presence of a developed micro- and mesoporous structure (total porosity of at least 70–80%) with a specific surface area of more than 200–500 m^2^/g, which ensures high access to active sorption centers.

### 5.3. Chemical Stability and Resistance

Cellulose-based sorbents exhibit significant chemical stability and effectiveness in various applications, particularly in water remediation. Most cellulose materials retain their sorption properties even when exposed to pollutants, such as heavy metals and organic compounds, for a long period of time [88]. To increase chemical stability, cellulose is often modified by introducing functional groups (such as carboxyl or sulfonic) into its structure, which increases its adsorption capacity and resistance to chemical interactions with pollutants. Their inherent properties, such as biodegradability and high surface area, make them suitable for adsorbing pollutants like CO_2_ and heavy metals. For instance, cellulose-based adsorbents have shown remarkable performance in removing heavy metals and dyes, with studies indicating that modified cellulose can enhance adsorption capacity significantly [89].

One of the key aspects considered in the article is the chemical stability of cellulose sorbents during long-term use. Modified cellulose materials demonstrated resistance to destructive factors, such as pH changes and the presence of high concentrations of metal ions. The article presents data confirming that such sorbents retain up to 80% of their sorption capacity after multiple adsorption and desorption cycles [90].

The environmental sustainability of the new adsorbent is also assessed in [91]. Regeneration studies have shown that after five adsorption–desorption cycles, the material retains more than 80% of its initial adsorption capacity, which emphasizes its durability and the possibility of repeated use without a significant decrease in efficiency. The authors note that simple methods, such as washing with a weak acid or alkaline solution, can be used for regeneration.

One of the important aspects discussed in the article is the stability and durability of lignocellulosic adsorbents during repeated use [92]. The data show that after several adsorption–desorption cycles, the materials retain their sorption capacity at a level of 70–80%, which indicates their chemical resistance to changes in pH, ionic strength, and the presence of competing pollutants. Particular attention is paid to the issue of chemical resistance of lignin, which is part of lignocellulosic materials and helps protect the adsorbent from aggressive chemical conditions. Lignin also helps increase selectivity with respect to organic compounds.

The study [93] compiles a broad list of different cellulose-based adsorbents, including modified cellulose, cellulose beads, grafted cellulose, cellulosic composites, nano-cellulose, and hydrogels, showcasing their comparable metal removal capacities to commercial adsorbents. Most of the modified cellulose adsorbents demonstrated strong regeneration abilities, allowing them to be reused efficiently across multiple cycles, which is beneficial for both cost-effectiveness and sustainability. The findings suggest that char and activated carbon derived from inexpensive cellulosic sources could potentially replace costly commercial activated carbon in heavy metal remediation efforts.

In [94], the authors considered the use of cellulose-based sorbents for the effective removal of heavy metals from wastewater. Despite many advantages, the authors also pointed out the limitations of using cellulose sorbents. They noted that reuse of these materials can reduce their efficiency, and after three regeneration cycles, the efficiency of lead ion removal can drop to 70%. This emphasizes the need for additional processing and control of the sorbent properties during repeated use.

Thus, the presented data here demonstrate the high efficiency of such materials in removing both organic and inorganic pollutants, as well as their environmental safety and potential for repeated use. At the same time, further research and development of new hybrid materials can significantly expand the application of lignocellulosic adsorbents in various water purification systems.

## 6. Sorption Properties of Cellulose Sorbents

**Figure 2:**
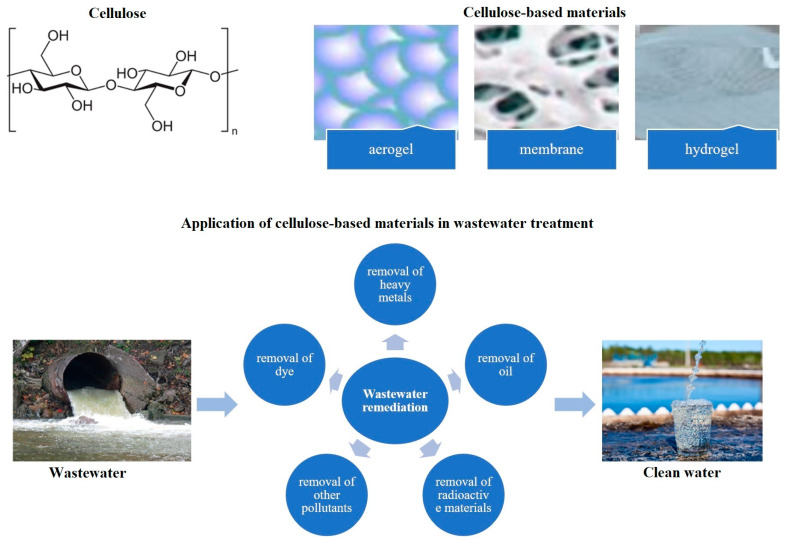
Application of cellulose-based materials in wastewater remediation.

The sorption properties of cellulose sorbents occupy a key place in research on wastewater treatment and the removal of various pollutants, including heavy metals, organic compounds, and radioactive elements (Figure 2). Being a natural polysaccharide with a high degree of availability, cellulose is actively used in the creation of sorbents due to its environmental safety, biodegradability, and ability for chemical modification.

Cellulose sorbents exhibit remarkable sorption properties, making them effective for removing contaminants such as heavy metals and organic liquids. Various studies highlight the efficiency of cellulose-based materials, including hydrogels, modified fibers, and nanocellulose sorbents, in adsorbing pollutants.

Seulgi Ji et al. investigated the efficiency of functionalized cellulose sorbents in removing hazardous organic liquids from aqueous solutions, emphasizing their environmental safety and potential for use in wastewater treatment [95]. The authors emphasized that, to improve the sorption properties of cellulose sorbents, they were modified using various functional groups. These changes significantly improved the interaction of the sorbent with organic pollutants such as toluene, benzene, and dioxane. The study showed that functionalized cellulose sorbents demonstrate high efficiency in removing various organic liquids. Specific data from the study indicated that, at an initial toluene concentration of 100 mg/L, the adsorption rate was up to 88% under optimal conditions of pH 7. The maximum adsorption capacity of the sorbent reached 200 mg/g, indicating its high efficiency in removing this pollutant. As for other organic liquids, the adsorption efficiency for benzene was 85% and, for dioxane, it was 82% under similar conditions. These figures confirm that modified cellulose sorbents can effectively adsorb hazardous organic substances from wastewater. The author also analyzed in detail the influence of various parameters on the sorption efficiency. It was found that an increase in the contact time between the sorbent and the pollutant to 120 min leads to a noticeable increase in the adsorption rate. The optimal pH values for the maximum removal of organic liquids ranged from 6 to 8, which also indicates the importance of controlling the experimental conditions. An equally important aspect considered in the article is the stability of functionalized cellulose sorbents. During repeated use tests, the sorbents retained their effectiveness, demonstrating a decrease in adsorption capacity of no more than 10% after three cycles. This confirms their economic feasibility and the possibility of repeated use in real conditions.

Similar results were obtained in the article of Nikiforova et al. [96], where the authors investigated the effect of the chemical modification of cellulose using 4-aminobenzoic acid on the sorption properties of cellulose sorbents for the removal of copper ions (Cu^2+^) from aqueous solutions. Studies have shown that the modified sorbents demonstrate high efficiency of Cu^2+^ ion adsorption. In particular, at an initial copper concentration of 100 mg/L, the adsorption degree reached 93%, which is significantly higher than that of unmodified cellulose, which showed a removal efficiency of about 55% under the same conditions. Under these conditions, the modified sorbent showed the highest results, achieving an adsorption capacity of up to 200 mg/g. The work also focused on the stability of the modified sorbents. In tests for multiple use, it was noted that after three adsorption cycles, the sorption efficiency decreased by no more than 5–7%, which confirms their high durability and possibility for use in real conditions.

Kumar et al. studied the synthesis and characterization of cellulose sorbents for the removal of heavy metal ions, such as Ni(II), Cu(II), and Pb(II), from aqueous solutions [97]. The paper described the methods for the synthesis of cellulose sorbents, including their modification with various reagents, such as carboxylic acid and amine compounds. During the experiments, it was found that the modified sorbents have a higher porosity and larger surface area, which contributes to the effective interaction with heavy metal ions. For example, at an initial concentration of copper ions of 100 mg/L, the adsorption degree reached 92%, which confirms the efficiency of cellulose sorbents in cleaning aqueous solutions. For lead ions, the removal efficiency was 95% and, for nickel, it was 89%. These data indicate that the modified sorbents were capable of effectively adsorbing heavy metal ions. After three adsorption and desorption cycles, the author noted that the efficiency of heavy metal ion removal decreased by no more than 10%, indicating the possibility of the repeated use of sorbents without significant loss of their sorption properties.

In [98], the authors provided a critical review of the use of modified cellulose as a bioadsorbent for the removal of divalent heavy metals ions such as cadmium (Cd^2+^), lead (Pb^2+^), and copper (Cu^2+^), from aqueous solutions. The authors discussed several methods of cellulose modification, including chemical and physical modification, which are aimed at increasing the adsorption properties of the material. For example, modification using glycidyl ether leads to the formation of additional functional groups that facilitate the binding of heavy metal ions. Studies have shown that modified sorbents are able to increase their sorption capacity. In particular, when cellulose is treated with glycidyl ether, the adsorption capacity for Cd^2+^ increases to 120 mg/g, which is 30% higher than that of unmodified cellulose. The article presented specific data on the sorption characteristics of various modified cellulose sorbents. For lead (Pb^2+^) ions,, the removal efficiency was shown to reach 95% at an initial concentration of 100 mg/L and a pH of 6–7. Similarly, sorbents modified with amino compounds demonstrated an efficiency of 92% for copper (Cu^2+^) ions under the same conditions. These figures confirm the high efficiency of modified cellulose sorbents in the purification process.

In [99], the authors reviewed current advances in the development of sustainable biomass-based adsorbents for the removal of inorganic toxic contaminants from wastewater. The authors discussed various types of biomass, such as agricultural waste, wood, and other plant materials, that can be used to create adsorbents. The paper presented many examples of the use of biomass as adsorbents for the removal of inorganic contaminants, such as lead (Pb^2+^), cadmium (Cd^2+^), and mercury (Hg^2+^). For example, rice husk-based adsorbents showed a lead removal efficiency of 98% at an initial concentration of 100 mg/L. For coconut charcoal-based adsorbents, the cadmium removal efficiency reached 95%, confirming their potential in wastewater treatment. It was found that the optimal pH value for lead ion removal is 6–7, which corresponds to neutral conditions. Contact time plays an important role: increasing the time to 120 min leads to an increase in the degree of toxic metal removal, which is confirmed by data in achieving a 90% cadmium removal efficiency. Studies have shown that, after several adsorption and desorption cycles, the heavy metal removal efficiency remains high, with a loss of no more than 5–10%.

Vokurova et al. studied the effect of various methods of preparing flax fiber-based sorbents on their functional properties, in particular, their sorption characteristics [100]. The article discussed several methods for preparing cellulose sorbents, including physical modification, chemical modification, and the use of composite materials. For example, with physical modification, it was found that treating flax fiber with steam at a temperature of 120 °C for 60 min leads to a significant improvement in its sorption properties due to changes in its structure and an increase in porosity. When using sodium acetate as a modifier, sorbents were able to adsorb up to 92% of cadmium (Cd^2+^) ions at an initial concentration of 100 mg/L. This high degree of removal confirms the effectiveness of chemical modification in increasing the interaction of the sorbent with pollutants. In addition, the author noted that the sorbents prepared using the combined method (physical + chemical modification) demonstrated improved properties. For example, such sorbents were able to adsorb 88% of lead (Pb^2+^) ions and 85% of copper (Cu^2+^) ions from solutions, indicating a significant increase in their functionality.

Cellulose, the most abundant natural polymer, possesses excellent hydrophilicity, chemical versatility, and biodegradability, making it an ideal base for hydrogel formation. By crosslinking cellulose chains or integrating functional groups, hydrogels can be engineered to exhibit superior sorption properties for a diverse range of contaminants. These materials leverage their three-dimensional porous networks to achieve high water uptake, improved contaminant trapping efficiency, and selective adsorption capabilities. Moreover, cellulose biomass-derived hydrogels capitalize on renewable feedstocks, aligning with the principles of a circular economy.

Recent advancements in the modification of cellulose-based hydrogels have expanded their application potential. For instance, in [101], the authors considered the sorption properties of cellulose hydrogels, emphasizing their potential in wastewater treatment. Cellulose-based hydrogels demonstrated the ability to adsorb up to 95% of lead (Pb^2+^) ions under optimal conditions, which was recorded at a pH from 5 to 7. In addition, the removal of cadmium (Cd^2+^) ions was about 85% at an initial concentration of 100 mg/L. The adsorption efficiency of copper (Cu^2+^) ions also reached 90% at a pH of 6, which confirmed the significant influence of environmental conditions on the sorption process. Regarding organic pollutants, the results showed that cellulose hydrogels can effectively remove 80–90% of dyes, such as methylene blue and PO-19 dye, at an initial concentration of about 50 mg/L. The maximum removal efficiency was achieved at a contact time of about 120 min, indicating the importance of optimizing this parameter.

In another study, the authors considered the creation and application of hydrogels based on cellulose biomass for wastewater treatment [102]. The authors described the process of synthesis of hydrogels based on cellulose biomass, which included the use of various modifiers, such as acrylic acid and other polymerizable compounds. The synthesis was carried out using free-radical polymerization methods, which allows for obtaining structures with high porosity and adsorption properties. It was found that such hydrogels have a maximum water-holding capacity reaching 3000% of the dry hydrogel weight, which makes them effective for use in conditions where a high degree of water absorption is required. The work also emphasized the effectiveness of hydrogels for removing pollutants from wastewater. In particular, the authors reported the results of experiments where cellulose-based hydrogels demonstrated the ability to remove heavy metal ions such as lead (Pb^2+^), cadmium (Cd^2+^), and copper (Cu^2+^). At an initial concentration of Pb^2+^ ions (100 mg/L), the removal efficiency was 92%; while for Cd^2+^ and Cu^2+^ this figure reached 89% and 91%, respectively. These data indicate the high adsorption characteristics of hydrogels, which make them potentially useful for wastewater treatment.

Oil spills and petroleum-based pollution present severe environmental challenges, threatening marine and terrestrial ecosystems, biodiversity, and water quality. Traditional cleanup techniques, such as chemical dispersants and mechanical methods, often fall short due to their limited efficiency, high costs, and potential for secondary contamination. This has driven research into innovative, eco-friendly solutions for oil spill remediation. Porous materials were developed based on cellulose nanofibrils that can be used as environmentally friendly sorbents for cleaning up oil and petroleum spills [103]. One of the key aspects of the study is the development of porous materials using cellulose nanofibrils (CNF). The article describes various strategies for synthesizing porous structures, including freezing and sublimation of water (freeze-drying), which allows for creating lightweight, porous structures with high sorption capacity, and physical crosslinking and chemical modification to improve mechanical and adsorption characteristics. In addition, the authors drew attention to the possibility of increasing the hydrophobicity of the material using chemical reagents or surfactants. The article presents the results of experiments showing that sorbents based on cellulose nanofibrils can effectively absorb oil and other hydrocarbon pollutants. In experiments, sorbents based on CNF showed a sorption capacity of up to 50–70 g of oil per 1 g of sorbent, which significantly exceeds the performance of traditional materials, such as synthetic polymers. It was also shown that such sorbents can be used repeatedly without a significant decrease in their efficiency. The authors also emphasized the low cost and availability of cellulose nanofibrils, which allows for the use of such sorbents not only in major accidents, but for routine cleaning of small oil and petroleum product spills, making this approach especially attractive for environmentally sensitive regions.

Cellulose, the most abundant natural polymer, exhibits remarkable versatility in material science due to its unique supramolecular structure and interaction with water. The hierarchical arrangement of cellulose chains into microfibrils and, subsequently, into crystalline and amorphous regions, profoundly influences its physicochemical properties, including water adsorption and swelling behavior. Recent studies have explored these structural variations to design cellulose materials with superior performance in applications such as pollutant removal, water retention, and controlled release systems. By elucidating how supramolecular changes affect water interaction, researchers aim to unlock new potential for cellulose in sustainable material science.

For instance, Grunin et al. studied the molecular structure of cellulose and its hydrophilic properties during water sorption [104]. The work focused on how changes in the supramolecular structure of cellulose affect its interaction with water and, accordingly, its adsorption characteristics. It was found that cellulose consists of microfibrils that are formed due to hydrogen bonds between molecules. During water sorption, the structure of cellulose undergoes changes, which leads to an increase in its hydrophilicity. During the experiments, the author recorded that, when cellulose is moistened, its structure changes, which allows for an increase in the availability of active sites for binding water molecules. The paper showed that the hydrophilic properties of cellulose depend on its supramolecular organization. According to the experiments, when cellulose is moistened, its moisture content can reach 12–15% of the dry material weight, which indicates a high degree of water adsorption. For cellulose samples with different degrees of polymerization, it was noted that longer polymer chains promote better water retention, while shorter chains lead to decreased hydrophilicity. The authors also studied the effect of temperature and time on the sorption process. It was noted that increasing the temperature to 30 °C promoted an increase in the rate of water adsorption. For example, during the first 30 min of sorption, there is a significant increase in the water-holding capacity of cellulose, which is confirmed by data showing that the rate of water adsorption reaches 70% of the maximum level during this period. The authors also discussed the durability of cellulose materials and their ability to retain their hydrophilic properties during long-term use. Experiments have shown that, after several cycles of wetting and drying, cellulose retains its structure, and the level of water retention remains at 10–12%. This indicates the possibility of reusing cellulose in systems requiring water absorption.

The adsorption of water by cellulose is primarily governed by its inherent hydrophilicity, which arises from the abundance of hydroxyl groups (-OH) in its structure. These hydroxyl groups readily form hydrogen bonds with water molecules, driving the hydration process. Based on the results of the literature review, probable mechanisms of sorption of heavy metal ions on cellulose sorbents are proposed. The results of the analysis of the studies allowed us to assume a general scheme of interaction between the components of the systems as follows:

- Physical sorption (electrostatic interaction):

Cellulose-OH (sorbent) + Me^2+^ ⇌ Cellulose-OH⋅⋅⋅Me^2+^ (1)

Here, the metal ion (Me^2+^) is retained on the surface of cellulose by weak electrostatic interactions.

- Ion exchange:

Cellulose-COOH + Me^2+^ → Cellulose-COO-Me^+^ + H^+^ (2)

In this reaction, the metal ion replaces the hydrogen ion located on the carboxyl group of cellulose.

- Complexation (on modified cellulose):

If cellulose is modified with amino groups (-NH_2_), the following complexation reaction is possible:

Cellulose-NH_2_ + Me^2+^ → [Cellulose-NH_2_⋅Me^2+^] (3)

The metal ion coordinates with the nitrogen atom in the amino group, forming a strong complex.

- Modification of cellulose using phosphate groups:

When phosphating cellulose, the following complex with a metal ion can be formed:

Cellulose-PO_4_^2−^ + Me^2+^ → Cellulose-PO_4_^2−^⋅Me^2+^ (4)

Phosphate groups help to increase sorption capacity by forming strong complexes with metal ions.

- Chemisorption (coordination of metal ions with functional groups):

Cellulose-OH + Me^2+^ → Cellulose-O-Me^+^ (5)

The metal ion coordinates with the hydroxyl group of cellulose to form a bond, which is an example of chemisorption.

Thus, cellulose-based sorbents hold remarkable potential for sustainable water treatment applications due to their renewable origin, biocompatibility, and tunable chemical structure. By leveraging diverse modification techniques—including hydrogel formation, functionalization with specific chemical groups, and incorporation into nanocomposites—researchers have significantly enhanced their adsorption efficiency for a wide range of contaminants, including heavy metal ions, hazardous organic compounds, and oil pollutants. Despite their promise, challenges remain in optimizing the regeneration processes, enhancing chemical stability under extreme conditions, and improving scalability for industrial applications. Future research should emphasize hybrid and composite materials to further boost performance and explore novel modification methods, such as radiation-induced techniques, to expand the applicability of cellulose-based sorbents in advanced water purification systems.

Nanocellulose sorbents represent a significant class within cellulose sorbents. These materials, created on the basis of nano-sized cellulose fibers, represent a real revolution in the field of water purification. Their unique properties—high specific surface area, biodegradability and the ability to be chemically modified—open up new horizons for scientists and engineers. They are capable of not only dealing with traditional pollutants, such as heavy metals or organic substances, but solving more complex problems, such as removing microplastics and toxic compounds [105,106,107].

This review indicates that modified nanocellulose, particularly cellulose nanofibers (CNFs) and cellulose nanocrystals (CNCs), shows significant potential for the adsorption of toxic heavy metal ions from water due to their large surface area and biocompatibility. The numerous surface hydroxyl groups on nanocellulose allow for various modifications, enhancing their ability to adsorb different heavy metals effectively. This suggests that the results likely demonstrate improved adsorption capacities [108].

This review highlights that nanocellulose-based materials are effective in removing a wide range of water contaminants, including heavy metals, dyes, drugs, pesticides, pharmaceuticals, and microbial cells. Nanocellulose can be produced in multiple forms, such as colloidal solutions, films, membranes, and hydrogels. Each form has specific applications in water treatment, showcasing the adaptability of nanocellulose materials for different treatment methods. This review outlines several methods employed for pollutant removal, including adsorption, filtration, and advanced techniques like reverse osmosis (RO) and electrofiltration. This review emphasizes the potential for processing cellulose into commercial products, which can enhance the use of nanocellulose as adsorbents and catalysts in water treatment applications [109].

Nanocellulose sorbents utilize various mechanisms, such as adsorption, electrostatic attraction, and chelation, to effectively bind heavy metal ions and dye pollutants. For instance, modified CNCs demonstrated a significant adsorption capacity for Cu^2+^ ions [110]. Nanocellulose fibers (NCFs) were successfully converted into cellulose nanocrystals (CNCs) and modified with diethylenetriamine-pentaacetic acid (DTPA) to create DTPA–CNCs, which were then tested for their ability to adsorb Cu^2+^ ions from water. The study found that several factors influenced the adsorption behavior, including pH levels, contact time, dosage of adsorbent, and the initial concentration of Cu^2+^ ions in the solution. The optimal conditions for maximum Cu^2+^ adsorption was determined to be a contact time of 2 h, a temperature of 35 °C, and a pH of 3. Under these optimal conditions, the maximum adsorption capacity of the modified CNCs was found to be 94.5 mg/g, indicating a significant improvement compared to unmodified NCFs due to the presence of functional groups on the DTPA–CNCs. The adsorption isotherm data best fit the Langmuir model, suggesting a monolayer adsorption on a surface with a finite number of identical sites.

The paper found that the structure of metal ions adsorbed onto cellulose nanocrystals (CNCs) changes with varying carboxylate density. Specifically, increasing the carboxylate density from 740 to 1100 mmol/kg transitioned the adsorption from a monolayer to a multilayer structure. The monolayer was modeled as a Stern layer surrounding the CNC nanoparticles, while the multilayer was described as a diffuse layer on top of the Stern layer. Within the Stern layer, the maximum ion density increased significantly from 1680 to 4350 mmol of Rb^+^ per kg of CNC as the carboxylate density increased. The paper highlighted that CNCs utilize multiple mechanisms, including electrostatic attraction and the chaotropic effect, to effectively adsorb ions of different valencies. Understanding the spatial organization of the adsorbed metal ions can help optimize the design of cellulose-based sorbents, enhancing their uptake capacity and selectivity for separation applications.

Studies have shown that the adsorption efficiency of nanocellulose can be optimized through various parameters, including pH, contact time, and concentration of pollutants. The nanocomposite developed in the study is notable for its exceptional swelling capacity, which allows it to absorb contaminants effectively. This property was enhanced through careful optimization of the nanocellulose content and the use of a biodegradable crosslinker, improving the composite’s mechanical and thermal stability. The primary pollutant studied in this publication was methylene blue, a dye commonly found in textile industry wastewater. The composite demonstrated a high adsorption capacity for this dye, underscoring its potential as a viable solution for industrial effluent treatment. The adsorption kinetics often follow models like Langmuir and Freundlich, indicating the multilayer adsorption processes. By focusing on agricultural waste as the raw material, the study not only provides an effective wastewater treatment method but contributes to sustainable development by promoting waste reuse [111].

Thus, while nanocellulose sorbents show great promise in water treatment, challenges remain in scaling up production and ensuring consistent performance across different environmental conditions.

## 7. Prospects for the Use of Sorbents Based on Cellulose

Cellulosic sorbents have a wide range of applications in water purification, removal of various pollutants, and toxic substances, including oil and oil products, microplastics, dyes, pesticides, and other organic pollutants. Their unique properties allow for effective adsorption of contaminants, particularly pharmaceuticals, heavy metals, and dyes, making them valuable in water remediation and other applications.

Cellulose and its derivatives have a high capacity for hydrocarbon adsorption, which makes them suitable for water purification from oil contaminants. The paper shows that the functionalization of cellulose sorbents with hydrophobic groups significantly improves their efficiency in removing oil products [112,113]. The papers consider the use of cellulose-based aerogels for water purification from oil spills. The results of the study in the articles reviewed show that the obtained cellulose aerogels have high porosity from 91% to 99%, which increases the adsorption capacity [114,115].

Microplastics are a serious environmental threat, and cellulose sorbents, due to their porous structure and surface modification capabilities, can effectively remove microplastic particles from water. The publication of Zhuang et al. showed that cellulose can be used to effectively filter microplastics, with good sorption properties at concentrations of up to 1000 parts per million (ppm) [116].

In [117], the authors studied the use of cellulose adsorbents for the extraction and recovery of pharmaceutical residues from water. The article described the methods of synthesis of cellulose adsorbents, including their modification in order to improve the sorption properties. In particular, the authors used the method of chemical modification, which improved the interaction of the adsorbent with pharmaceutical residues. The paper presented the results of experiments on the removal of various pharmaceutical compounds, such as ibuprofen, diclofenac, and antibiotics. It was found that the optimal sorption capacity of cellulose-based adsorbents reaches 150 mg/g for ibuprofen at an initial concentration of 1000 μg/L. For other compounds, such as diclofenac, the maximum sorption capacity was 120 mg/g. This confirms the high efficiency of cellulose adsorbents in the removal of pharmaceutical residues from the aqueous medium. Studies have shown that extraction conditions, such as pH, temperature, and contact time, significantly affect the sorption efficiency. The optimum pH value for removing pharmaceutical residues is around 6–7, which corresponds to neutral conditions. A contact time of 30–60 min provides maximum sorption capacity, while increasing the temperature to 30 °C also helps improve the extraction results. Experiments have shown that after 5 cycles of adsorption and desorption, the removal efficiency of pharmaceutical residues decreased by only 10–15%, indicating their durability and economic feasibility for practical application.

Taherpoor et al. described the synthesis of functionalized cellulose nanocomposites incorporating various active components, such as nanometal oxide, which improved their adsorption properties [118]. The article indicated that modification of cellulose with 20 wt.% of nano zinc oxides (ZnO) led to an increase in the porosity of the adsorbent and improved its mechanical properties, which in turn contributed to more efficient adsorption of pharmaceutical residues. As a result of the experiments, it was found that the functionalized nanocomposites demonstrate high efficiency in the removal of various pharmaceutical compounds, including diclofenac and paracetamol. At an initial concentration of 1000 μg/L of diclofenac, the maximum sorption capacity was 200 mg/g, which confirms the high performance of this adsorbent. For paracetamol, the maximum sorption capacity was 180 mg/g. The optimum pH for removing pharmaceutical contaminants was found to be 6.5–7, which corresponds to a neutral environment. A contact time of 60–120 min was determined to be optimal for achieving maximum adsorption capacity. The ambient temperature also plays an important role: increasing the temperature to 30 °C leads to an increase in the adsorption rate.

The textile and other industries often use a large number of dyes that are stable and toxic to the environment. Due to its porous structure and possibility for modification, cellulose can effectively remove dyes from wastewater. For example, modification of cellulose sorbents using amino groups significantly improves their ability to sorb organic pollutants, including dyes [119,120,121].

In the food industry, modified cellulose serves as smart packaging materials that monitor food quality, enhancing safety and shelf life [122]. In agrochemistry, cellulose’s ability to adsorb heavy metals from wastewater highlights its potential for environmental remediation [123]. Furthermore, cellulose-based materials are pivotal in carbon capture technologies, acting as effective adsorbents for CO_2_ [124].

Thus, cellulose sorbents represent a promising solution for water purification from various pollutants. The development of new methods for their modification significantly expands their potential in environmental, food, and pharmaceutical applications, which opens up new opportunities for use in various industries and environmental protection.

## 8. Conclusions

Cellulose-based sorbents, due to their unique physical and chemical properties, are a promising basis for efficient wastewater treatment from heavy metals and other pollutants. This analysis shows that modified cellulose materials have high sorption capacity, are environmentally friendly, and stable, which makes them competitive with other modern technologies.

Chemical modification methods, such as carboxylation, amination, and graphene treatment, can significantly improve the sorption properties of cellulose. These approaches help increase the number of functional groups on the sorbent surface, which enhances its ability to bind pollutants. Combined methods that combine several technologies make it possible to obtain materials with unique characteristics: high stability, versatility, and resistance to extreme operating conditions.

The morphological and porous properties of sorbents play a key role in their effectiveness. Increasing the specific surface area, optimizing the pore size, and increasing the degree of crystallinity of cellulose have a positive effect on the sorption kinetics and maximum sorption capacity. Case studies confirm that targeted changes in morphological characteristics can improve material performance.

The chemical stability and durability of cellulose sorbents have also been confirmed in various studies. Such materials are resistant to aggressive operating conditions, including high temperatures, pH changes, and repeated regeneration cycles, which makes them particularly attractive for industrial applications.

The undeniable advantages of cellulose sorbents are their biodegradability, renewability, cost-effectiveness, and availability of raw materials. However, there are certain challenges, including optimization of modification technology, reduction of production costs, and increased scalability. These aspects require further attention to improve the practical applicability of cellulose sorbents.

Thus, cellulose-based sorbents are promising materials for implementation in environmentally friendly water treatment technologies. Future research should focus on the creation of innovative modification methods, study of sorption mechanisms, and development of effective solutions for the use of such sorbents on an industrial scale.

## Figures and Tables

**Table 1:** Main properties of cellulose.

Property	Description	Value for Sorption	Sources
Chemical structure	A linear polysaccharide consisting of β-d-glucose linked by β-(1→4)-glycosidic bonds.	It forms crystalline and amorphous areas. Amorphous regions provide better accessibility of hydroxyl groups, crystalline zones provide mechanical stability.	[4]
Crystallinity	Cellulose has both crystalline and amorphous zones. The degree of crystallinity varies depending on the source of cellulose.	Crystalline zones improve mechanical strength but reduce reactivity, while amorphous zones promote adsorption.	[5]
Porous structure	Depending on the treatment, cellulose can form porous structures.	Porosity increases the specific surface area, which improves sorption properties by increasing contact with pollutants.	[13]
Mechanical strength and stability	High strength and resistance to mechanical damage, due to crystalline zones and hydrogen bonds.	Mechanical strength is important for the stability of sorbents during operation, especially during filtration and dynamic sorption processes.	[14]
Presence of hydroxyl groups (-OH)	Cellulose contains a large number of active hydroxyl groups on the surface.	Hydroxyl groups provide the possibility of chemical modification, which increases the sorption capacity.	[15]
Hydrophilicity	High affinity for water due to hydroxyl groups.	Hydrophilicity promotes adsorption of polar pollutants, such as heavy metals and some organic compounds.	[15]
Stability in aggressive environments	Cellulose is resistant to weak acids and alkalis, but sensitive to strong acids and alkalis.	Resistance to aggressive environments allows for the use of cellulose sorbents in industrial water purification processes.	[16]
Modifiability	Easily modified through esterification, carboxylation, acetylation, and other chemical reactions.	Chemical modification allows for the creation of specialized sorbents for target pollutants, such as heavy metals or organic compounds.	[17,18]
Specific surface area	Increases with the transition to nano-sized forms, such as nanocrystals and nanofibrils.	High specific surface area contributes to an increase in the number of available sorption centers.	[19]
Environmental safety	Cellulose is a renewable natural polymer, biodegradable, and environmentally friendly.	Environmental safety makes cellulose an excellent basis for the development of “green” technologies for water purification and cleaning contaminated soils.	[20,21]

**Table 2:** Advantages and disadvantages of cellulose modification methods.

Modification Method	Advantages	Disadvantages	Process Conditions	Sorbent Yield	Results	Sources
Carboxylation of cellulose	1. High adsorption capacity for metal ions. 2. Simplicity of the process.	1. The use of acids may lead to corrosion of equipment. 2. Possibility of formation of by-products.	Temperature: 60–80 °C, pH: 3–5, time: 2–4 h	80% of the initial material mass	High adsorption capacity for metal ions (e.g., Pb^2+^)—81.3 mg/g	[68]
Amination of cellulose	1. High efficiency for heavy metal removal. 2. Modified sorbent is resistant to repeated use.	1. The process requires the use of toxic reagents. 2. Expensive reagents for amination.	Temperature: 90 °C, pH: 8, time: 5 h	85% of the initial material mass	Heavy metal removal efficiency—98% for Cd^2+^	[69]
Graphene modification of cellulose	1. Increased porosity and extended diffusion path. 2. Improved sorption capacity for organic pollutants.	1. Complexity of synthesizing graphene materials. 2. High cost of graphene additives.	Temperature: 100 °C, time: 4 h	70% of the initial material mass	~100% removal of heavy metals	[70]
Mechanical activation of cellulose	1. Fast processing. 2. Increased material stability during operation.	1. Reduction in porosity can lead to a decrease in sorption capacity. 2. Need for special equipment	Temperature: 50 °C, time: 1 h	90% of the initial material mass	Reduction in porosity, but increase in sorbent stability	[71]
Radiation-induced grafting	1. High adsorption capacities 2. Good mechanical and thermal stability	1. High cost of equipment and energy consumption. 2. Possibility of uncontrolled destruction of cellulose with excessive radiation dose. 3. Further optimization of methods is required for large-scale application.	Processing is usually carried out at room temperature. Gamma rays or electron beams with energies of 2–10 MeV are used.	About 70–90%, depending on the radiation dose and the type of associated reactions	Adsorption of organic dyes increases by 30–50% compared to unmodified cellulose.	[72]

## Data Availability

Not applicable.
